# Extracellular domain shedding influences specific tumor uptake and organ distribution of the EGFR PET tracer ^89^Zr-imgatuzumab

**DOI:** 10.18632/oncotarget.11827

**Published:** 2016-09-02

**Authors:** Martin Pool, Arjan Kol, Marjolijn N. Lub-de Hooge, Christian A. Gerdes, Steven de Jong, Elisabeth G.E. de Vries, Anton G.T. Terwisscha van Scheltinga

**Affiliations:** ^1^ Department of Medical Oncology, University of Groningen, University Medical Center Groningen, Groningen, The Netherlands; ^2^ Department of Clinical Pharmacy and Pharmacology, University of Groningen, University Medical Center Groningen, Groningen, The Netherlands; ^3^ Department of Nuclear Medicine and Molecular Imaging, University of Groningen, University Medical Center Groningen, Groningen, The Netherlands; ^4^ Department of Roche Pharma Research and Early Development, Roche Innovation Center Zürich, Schlieren, Switzerland

**Keywords:** imgatuzumab, EGFR, ^89^Zr, immunoPET, shedding

## Abstract

Preclinical positron emission tomography (PET) imaging revealed a mismatch between *in vivo* epidermal growth factor receptor (EGFR) expression and EGFR antibody tracer tumor uptake. Shed EGFR ectodomain (sEGFR), which is present in cancer patient sera, can potentially bind tracer and therefore influence tracer kinetics. To optimize EGFR-PET, we examined the influence of sEGFR levels on tracer kinetics and tumor uptake of EGFR monoclonal antibody ^89^Zr-imgatuzumab in varying xenograft models. Human cancer cell lines A431 (EGFR overexpressing, epidermoid), A549 and H441 (both EGFR medium expressing, non-small cell lung cancer) were xenografted in mice. Xenografted mice received 10, 25 or 160 μg ^89^Zr-imgatuzumab, co-injected with equal doses ^111^In-IgG control. MicroPET scans were made 24, 72 and 144 h post injection, followed by biodistribution analysis. sEGFR levels in liver and plasma samples were determined by ELISA. ^89^Zr-imgatuzumab uptake in A431 tumors was highest (29.8 ± 5.4 %ID/g) in the 160 μg dose group. Contrary, highest uptake in A549 and H441 tumors was found at the lowest (10 μg) ^89^Zr-imgatuzumab dose. High ^89^Zr-imgatuzumab liver accumulation was found in A431 xenografted mice, which decreased with antibody dose increments. ^89^Zr-imgatuzumab liver uptake in A549 and H441 xenografted mice was low at all doses. sEGFR levels in liver and plasma of A431 bearing mice were up to 1000-fold higher than levels found in A549, H441 and non-tumor xenografted mice. ^89^Zr-imgatuzumab effectively visualizes EGFR-expressing tumors. High sEGFR levels can redirect ^89^Zr-imgatuzumab to the liver, in which case tumor visualization can be improved by increasing tracer antibody dose.

## INTRODUCTION

Overexpression and mutations of epidermal growth factor receptor (EGFR) are associated with tumor cell growth, differentiation, proliferation, apoptosis and cellular invasiveness [1]. Clinical treatment options for *KRAS* wild-type EGFR in head and neck squamous cell carcinoma (HNSCC) and metastatic colorectal cancer (mCRC), encompass monoclonal antibodies (mAbs) cetuximab and panitumumab. Mutant EGFR expressing tumors, including non-small cell lung cancer (NSCLC), are treated with the tyrosine kinase inhibitors erlotinib and gefitinib [2–4].

EGFR-targeted therapy might be improved by optimizing antibody-dependent cell-mediated cytoxicity (ADCC) responses. Imgatuzumab (GA201) is a novel humanized anti-EGFR IgG1 isotype mAb, glycoengineered for enhanced ADCC, as well as inhibiting ligand-dependent signaling of EGFR. Imgatuzumab recognizes human EGFR and is not cross-reactive with murine EGFR [5]. It showed superior *in vivo* efficacy compared to cetuximab and non-glycoengineered imgatuzumab in both *KRAS*-mutant and *KRAS*-wild type tumor models. In phase 1 studies imgatuzumab demonstrated promising efficacy in heavily pretreated patients with advanced EGFR-positive solid tumors and *KRAS*-mutant EGFR-positive advanced colorectal cancer [6, 7].

Whole body determination of EGFR expression in lesions using molecular imaging could support decision making during clinical development and clinical practice. Antibody biodistribution can be visualized by labeling mAbs with the PET isotope Zirconium-89 (^89^Zr, t½ = 78.4 h). We previously developed and successfully tested ^89^Zr-labeled antibody-based tracers targeting human epidermal growth factor receptor 2 (HER2), vascular endothelial growth factor (VEGF) and HER3 in the preclinical and clinical setting [8–12]. ^89^Zr-labeled antibodies have also been developed for preclinical EGFR imaging [13–15]. Furthermore, clinical EGFR imaging has been performed with ^89^Zr-cetuximab in advanced colorectal cancer patient [16,17]. Additional clinical studies are currently ongoing for both ^89^Zr-cetuximab and ^89^Zr-panitumumab (ClinicalTrial.gov identifiers NCT01691391, NCT02117466 and NCT02192541).

Unfortunately, preclinical EGFR antibody-based imaging studies revealed a mismatch between *in vivo* EGFR protein expression and tumor tracer uptake [13, 17]. Many factors have been suggested for this mismatch, including perfusion rates, vascularity, vascular permeability, interstitial pressure and mAb plasma half-life [17]. Circulating HER2 extracellular domain (ECD) and trastuzumab are known to form complexes, which are swiftly cleared by the liver [18]. Underscoring the possibility of shed ECD to significantly influence kinetics of antibodies, shed HER2 serum levels over 500 ng/mL extensively influenced trastuzumab kinetics in patients at weekly doses of 100 mg trastuzumab [19]. For EGFR and EGFR-targeting mAbs this relation is less clear. However, some studies show extensive levels of circulating soluble EGFR extracellular domain (sEGFR) in patients and healthy volunteers [20]. sEGFR might therefore also influence kinetics and tumor uptake of tracer doses used for ^89^Zr-labeled EGFR targeting antibodies.

In order to optimize EGFR imaging and visualize EGFR expression *in vivo* we developed the EGFR PET tracer ^89^Zr-imgatuzumab and examined the influence of sEGFR on ^89^Zr-imgatuzumab tracer kinetics and tumor uptake in multiple xenograft models using microPET imaging.

## RESULTS

### ^89^Zr-imgatuzumab tracer development and quality control

Df-imgatuzumab conjugate bound up to 500 MBq ^89^Zr/mg Df-imgatuzumab at a radiochemical purity (RCP) ≥ 95% after radiolabeling, without further purification (Supplementary Figure S1A). SE-HPLC revealed absence of aggregates and fragments in Df-imgatuzumab conjugates. The immunoreactive fraction of Df-imgatuzumab conjugate was assessed by competition assay at 68.9 ± 6.3% compared to unmodified imgatuzumab (Supplementary Figure S1B). ^89^Zr-imgatuzumab was stable *in vitro*, the maximum observed decrease in RCP was from 99.4 ± 0.1% to 93.4 ± 0.8% in 0.5 M HEPES buffer pH 7.2 after 14 days at 37°C (Supplementary Figure S1C). All batches of ^89^Zr-imgatuzumab used as tracer had a RCP ≥ 95% by TCA precipitation, while ^111^In-IgG batches had a RCP ≥ 90% by ITLC.

### MicroPET imaging and biodistribution in different tumor models

**Figure 1:**
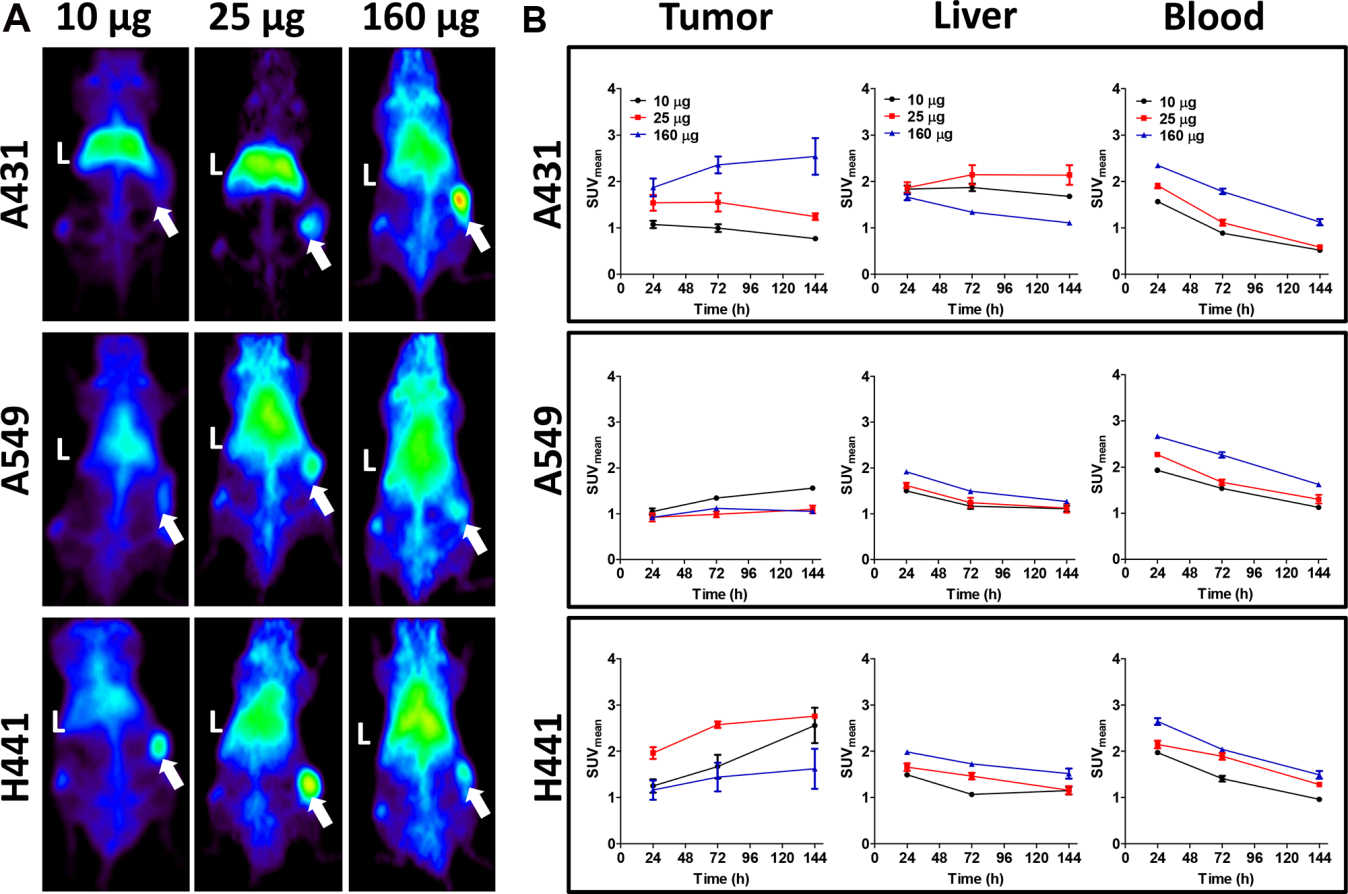
(**A**) Representative maximal intensity projections of microPET scans 144 h after tracer injection for 10, 25 and 160 μg doses of ^89^Zr-imgatuzumab in A431, A549 and H441 xenograft bearing mice. Livers are marked with L, while xenografts are marked with an arrow. (**B**) MicroPET quantification for tumor, liver and blood pool at 24, 72 and 144 h after tracer injection for 10, 25 and 160 μg tracer dose in A431, A549 and H441 xenograft bearing mice. Data are expressed as SUV_mean_ ± SEM.

MicroPET analysis showed preferential tumor uptake of ^89^Zr-imgatuzumab in A431, A549 and H441 xenografts models (Figure 1A). For A549 and H441, the highest ^89^Zr-imgatuzumab tumor uptake was observed for the 10 μg dose 6 days pi (Figure 1B), while higher doses lowered tumor uptake. An opposite pattern was observed in A431 tumors, where ^89^Zr-imgatuzumab tumor uptake increased with increasing tracer dose (Figure 1B). Blood levels of ^89^Zr-imgatuzumab were highest for the 160 μg tracer dose at day 1 pi for all tested models and decreased gradually over time (Figure 1B). ^89^Zr-imgatuzumab blood levels were significantly lower in A431 tumor bearing mice compared to both A549 and H441 at 10 μg and 25 μg (*P* < 0.001) and at 160 μg (A549: *P* < 0.01, H441: *P* < 0.05). High liver accumulation was observed in A431 compared to both A549 and H441 tumor bearing mice at 10 μg (*P* < 0.001) and at 25 μg (*P* < 0.01), but not in the 160 μg tracer dose group (Figure 1A, 1B).

**Figure 2:**
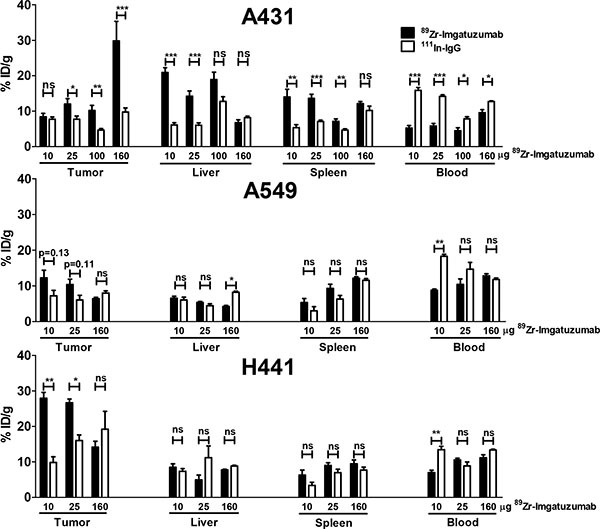
*Ex vivo* uptake data for tumor, liver, spleen and blood, presented as % ID/g ^89^Zr-imgatuzumab and ^111^In-IgG non-specific control, for 10, 25 and 160 μg doses in A431, A549 and H441 xenograft bearing mice

**Figure 3:**
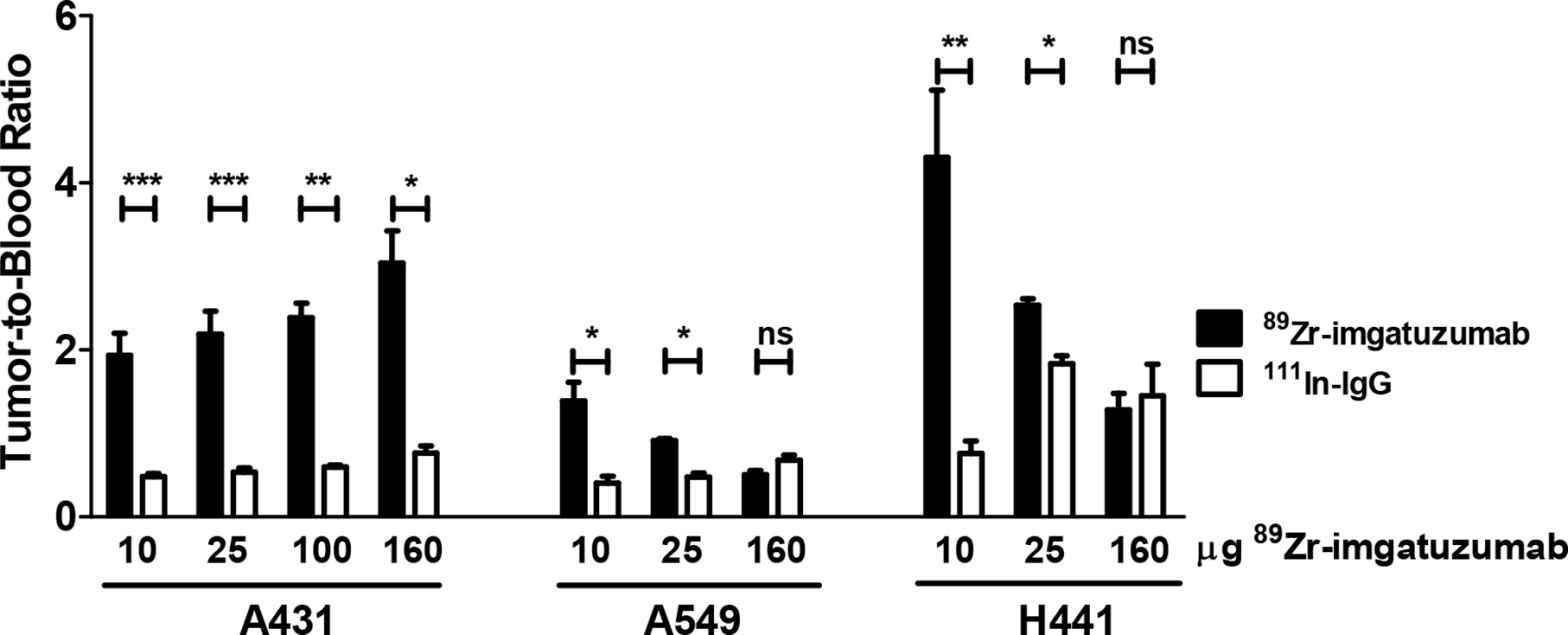
Tumor-to-blood ratios of ^89^Zr-imgatuzumab and ^111^In-IgG for indicated doses in A431, A549 and H441 xenograft bearing mice

Biodistribution studies showed that ^89^Zr-imgatuzumab uptake in A431 tumors was not different from ^111^In-IgG uptake in the 10 μg dose group (8.4 ± 1.0 vs. 7.7 ± 0.7%ID/g, *P* = 0.50), but after increasing the dose to 25, 100 and 160 μg, significant tumor uptake was observed. Highest A431 tumor uptake was observed at 160 μg tracer dose, with 29.8 ± 5.4 %ID/g for ^89^Zr-imgatuzumab, compared to 9.8 ± 1.1 %ID/g for ^111^In-IgG uptake (*P* < 0.05) (Figure 2). Tumor-to-blood ratios of all tracer doses in A431 tumor bearing mice were consistently higher for ^89^Zr-imgatuzumab compared to ^111^In-IgG, revealing absolute tracer uptake in A431 tumors was limited by blood pool availability of ^89^Zr-imgatuzumab (Figure 3). Tracer uptake in A549 in tumors was the highest for the 10 μg dose group at 12.2 ± 2.1%ID/g for ^89^Zr-imgatuzumab vs. 7.2 ± 1.5 %ID/g for ^111^In-IgG (*P* = 0.13), while tumor-to-blood values for 10 and 25 μg were significantly higher for ^89^Zr-imgatuzumab compared to ^111^In-IgG. The highest specific tumor uptake in H441 tumors was observed with 10 μg ^89^Zr-imgatuzumab (28.0 ± 1.6%ID/g) compared to ^111^In-IgG (9.8 ± 1.6%ID/g, *P* < 0.01), whereas at 160 μg ^89^Zr-imgatuzumab tumor uptake (14.2 ± 1.7%ID/g) was similar to ^111^In-IgG (19.2 ± 5.0%ID/g, Figure 2). Both A549 and H441 tumors showed target saturation at the 160 μg dose (Figures 2, 3). Tumor-to-blood ratios for both A549 and H441 showed a similar pattern to absolute uptake data, denoting uptake of ^89^Zr-imgatuzumab in these tumors was not limited by blood pool availability (Figure 3).

High liver uptake of 20.9 ± 1.3%ID/g was observed in mice bearing A431 tumors at 10 μg ^89^Zr-imgatuzumab, this was higher than for A549 (6.5 ± 0.5%ID/g, *P* < 0.0001) and H441 (8.5 ± 1.0%ID/g, *P* < 0.001) bearing mice at equal dose. Liver uptake in A431 bearing mice could be blocked by increasing the tracer antibody dose, as can be seen by the decreasing differences between ^89^Zr-imgatuzumab and ^111^In-IgG liver uptake (Figure 2). Specific spleen uptake of ^89^Zr-imgatuzumab was observed in all tumor models, which was saturable at higher doses, as shown by decreasing differences between ^89^Zr-imgatuzumab and ^111^In-IgG spleen uptake at higher tracer doses (Figure 2).

### EGFR expression analysis and immunohistochemistry

**Figure 4:**
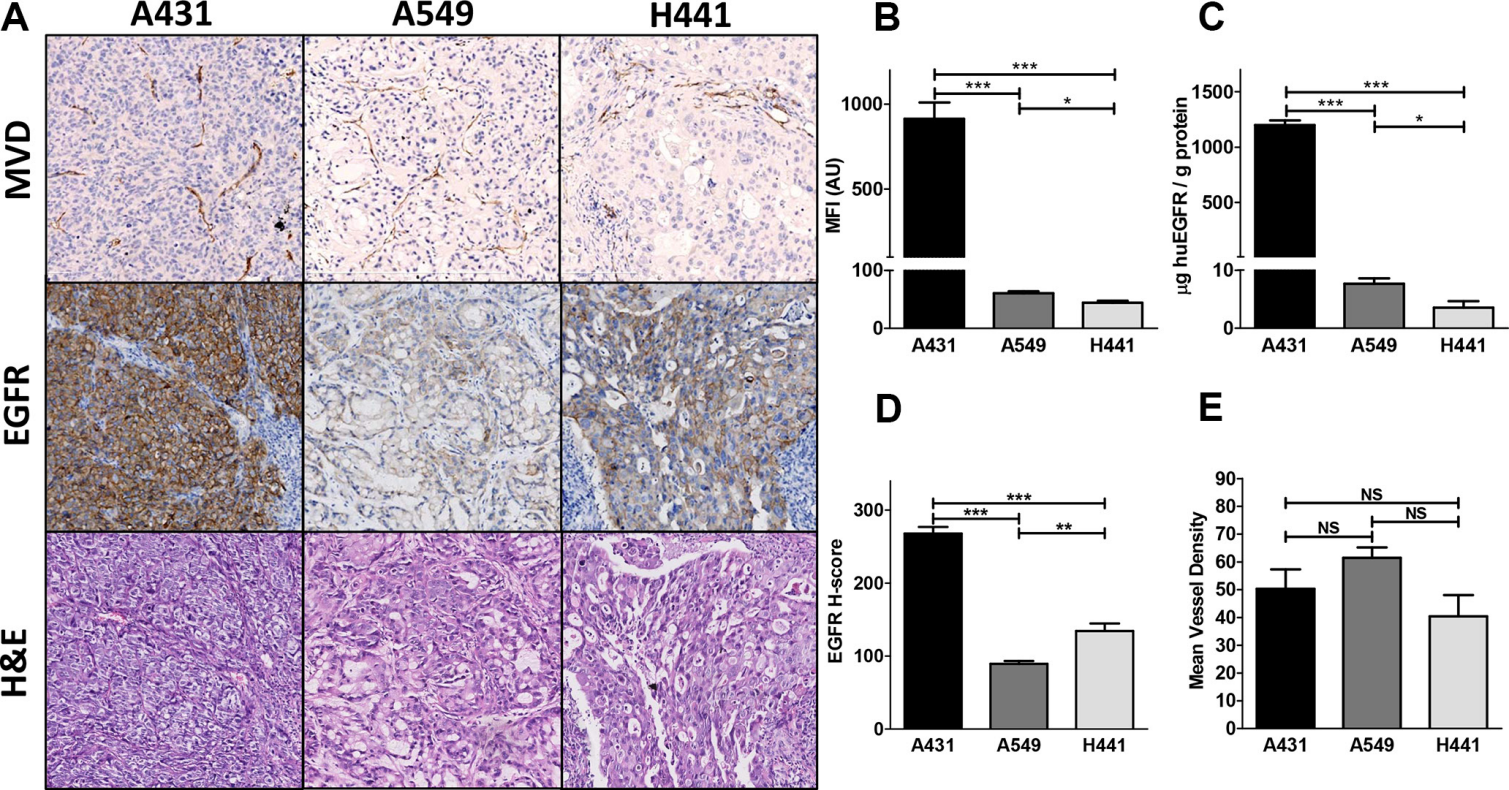
(**A**) *Ex vivo* tissue analysis. Mean vessel density (MVD), EGFR and hematoxylin and eosin immunohistochemical staining of A431, A549 and H441 tumors. (**B**) *In vitro* flow cytometric analysis of EGFR membrane expression in A431, A549 and H441 cells. (**C**) *Ex vivo* human EGFR extracellular domain levels in A431, A549 and H441 tumor tissue lysates. (**D**) EGFR histo-score (H-score) for A431, A549 and H441 tumors. (**E**) Mean vessel density score for A431, A549 and H441 tumors.

EGFR cell surface expressions *in vitro* by flow cytometric analysis (Figure 4B) and *ex vivo* by ELISA in tumor lysates (Figure 4C) were concordant. Expression level of EGFR per gram protein based upon ELISA was highest in A431, followed in declining order by A549 and H441 xenografts. All xenograft tumors expressed EGFR as determined by IHC (Figure 4A). Highest EGFR H-score was found in A431, followed by H441 and the least amount of staining was found in A549 xenografts (Figure 4A and 4D). A549 tumor cell density was markedly lower compared with A431 and H441 tumors (Figure 4A). Microvascular density was found to be similar in all three xenograft models (Figure 4E).

### sEGFR analysis of plasma, liver and cell lysates

**Figure 5:**
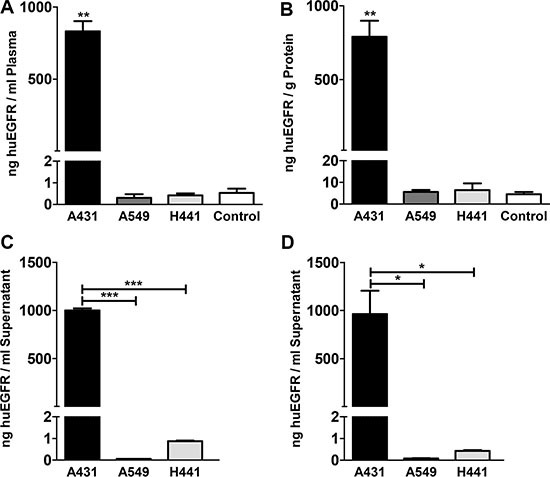
(**A**) Human EGFR extracellular domain concentration in plasma of A431, A549 and H441 xenograft bearing and non-tumor bearing control mice. (**B**) Human EGFR extracellular domain level in liver tissue lysates of A431, A549 and H441 xenograft bearing and non-tumor bearing control mice. (**C**) Human EGFR extracellular domain concentration in serum-free supernates of confluent cell cultures of A431, A549 and H441 cells. (**D**) Human EGFR extracellular domain concentration in serum-containing supernates of confluent cell cultures of A431, A549 and H441 cells, only statistical significances against A431 are shown for clarity.

ELISA showed high sEGFR levels (831 ± 71 ng/mL) in plasma of A431 tumor bearing, which were three magnitudes higher than observed in A549, H441 and non-tumor bearing control mice (*P* < 0.01). A549 and H441 sEGFR plasma levels did not deviate from background measurement in plasma of non-tumor bearing control mice (Figure 5A). sEGFR levels were also elevated at 790 ± 109 ng/g protein sEGFR in liver lysates of A431 xenograft bearing mice (*P* < 0.01), but not in A549 and H441 when compared to non-tumor bearing control mice (Figure 5B). To confirm the shedding potential of A431 *in vitro*, confluent cell cultures serum-free supernates were tested for sEGFR. sEGFR levels in A431 supernates (999 ± 21 ng/ml) were approximately 1000-fold higher (*P* < 0.001) compared to A549 and H441 (0.06 ± 0.004, respectively 0.87 ± 0.03 ng/ml) (Figure 5C). sEGFR in supernates from serum-containing media cultures showed a similar pattern and equal magnitude, indicating inhibition of proteolytical cleavage by serum did not influence the amount of sEGFR shedding in these cell lines (Figure 5D). Serum-free and serum-containing media controls were negative for sEGFR.

## DISCUSSION

This is the first paper showing tumor uptake of an EGFR-targeted antibody tracer can be affected by EGFR shedding. High levels of sEGFR acted as antigen sink, redirecting ^89^Zr-imgatuzumab to the liver, which reduced tumor uptake at lower tracer doses in A431 xenografts. Contrarily, in A549 and H441 xenografts models, lacking detectable EGFR shedding, low liver uptake was observed and tumor uptake could be saturated by ^89^Zr-imgatuzumab dose increments.

A431 xenografts showed the highest tumor uptake at the highest tracer dose used. Specific liver uptake of ^89^Zr-imgatuzumab was observed, which decreased with dose increments in A431 bearing mice. In previous preclinical EGFR antibody-based imaging studies revealing mismatch between xenograft EGFR expression and tracer uptake, a large role was postulated for vascularity and vascular permeability for tracer delivery in the tumor [13, 17]. Our results show that in addition to vascular differences between xenografts, circulating sEGFR can act as antigen sink and decrease tumor tracer uptake. Complexation of ^89^Zr-imgatuzumab and sEGFR, followed by liver sequestration as shown by ELISA, resulted in high ^89^Zr-imgatuzumab liver uptake, low blood pool availability and consequently relatively low uptake in A431 xenograft tumors at low tracer protein doses.

In contrast, in A549 and H441 xenografts low ^89^Zr-imgatuzumab liver uptake and sEGFR plasma levels were observed. In these models ^89^Zr-imgatuzumab tumor uptake was dose dependent, with highest uptake observed for low tracer protein doses, comparable to earlier findings for various antibody-based tracers targeting HER family members [8, 10, 15]. Higher ^89^Zr-imgatuzumab tumor uptake was found in H441 compared to A549 xenografts in our study, despite lower *in vitro* and *ex vivo* EGFR expression based upon ELISA. No difference in microvascular density was found between all models. Therefore, differences in ^89^Zr-imgatuzumab uptake in A549 and H441 are most likely explained by the lower EGFR expression based upon EGFR histoscore, combined with lower tumor cell density on H&E in A549. Furthermore, vascular permeability in the H441 is higher, as shown by the high accumulation of ^111^In-IgG control at higher doses, which was also seen in an earlier study [10]. Specific spleen uptake of ^89^Zr-imgatuzumab was observed in all tumor models, which decreased with ^89^Zr-imgatuzumab dose increments. Spleen targeting of ^89^Zr-imgatuzumab can be explained by its affinity, through glycoengineering, for the murine analog of the FcyRIIIA receptor, FcγRIV, on macrophages and monocytes [5] and resembles our experience with similarly glycoengineered HER3 mAb ^89^Zr-RG7116 [10].

Several isoforms of circulating sEGFR have been found in ovarian, metastatic breast and non-small cell lung cancer patients and healthy volunteers [21–24]. All sEGFR isoforms contain subdomain III of the EGFR extracellular domain, to which EGFR mAbs imgatuzumab, cetuximab and panitumumab bind [25, 26], but lack the transmembrane and cytoplasmic domains of the EGFR receptor [21, 22]. While cetuximab is known to inhibit sEGFR shedding *in vitro* (27), it is currently unknown whether imgatuzumab has potential to inhibit sEGFR shedding. sEGFR is commonly shed by proteolytic cleavage from human carcinoma cells expressing over 7 × 10^5^ EGFR receptors [27]. Of the present cell panel, only A431 is known to shed a ∼105 kDa sEGFR isoform, due to an aberrant transcript, as well as a 110 kDa sEGFR isoform [29–31]. Because of its aberrant sEGFR shedding potential, the A431 cell line was used to reliable induce human sEGFR shedding in a murine system and served as a model for the interaction between the endogenous sEGFR isoforms and EGFR monoclonal antibody tracers. Besides proteolytic cleavage, alternate splicing events in normal tissues result in mRNA transcripts encoding for 60-kDa, 80-kDa and 110-kDa sEGFR isoforms, that are the major source of sEGFR in patients and healthy volunteers [22].

sEGFR levels in A431 tumor-bearing mice were 837 ± 71 ng/mL plasma, corresponding to ∼2.5 sEGFR for each ^89^Zr-imgatuzumab molecule in circulation for the 10 μg tracer dose, reflecting a high degree of competition of sEGFR with tumor-bound EGFR. Increasing the ^89^Zr-imgatuzumab dose to 160 μg, mitigated sEGFR competition for tumor bound EGFR, resulting in higher tumor uptake. A limited number of preclinical imaging studies have included shed or circulating antigen measurements. c-MET tracer ^89^Zr-onartuzumab kinetics were not influenced by 14.1 ± 5.2 ng/mL per gram tumor shed c-MET in MKN-45 xenografts at high tracer doses of 64 to 128 μg [31]. However, similar to our results, high CA19.9 mAb ^89^Zr-5B1 liver uptake and decreasing tumor uptake over time was observed for in Colo205-Luc xenografts secreting large amounts CA19.9 (2500 ± 1033 U/mL), but not in DMS79 and BcPC3 xenograft bearing mice with no detectable serum CA19.9 [32]. Free prostate specific antigen (PSA) targeting ^89^Zr-5A10 showed a similar principle, liver uptake was 24.6 ± 4.9%ID/g for ^89^Zr-5A10 in LNCaP-AR xenograft bearing mice in presence of 18.35 ± 2.7 ng/mL free PSA in serum. Liver uptake was 2.5 fold lower for both ^89^Zr-5A10 in PSA-negative PC3 tumor bearing mice and ^89^Zr-IgG control in LNCaP-AR tumor bearing mice [33], confirming the ability of circulating antigen to direct antibody tracers to the liver.

In transgenic mice developing mutant and KRAS mutated NSCLC, murine endogenous plasma sEGFR and EGFR liver expression decreased after carcinogenesis [33]. Lower concentrations circulating sEGFR were also found in humans with NSCLC, when compared to healthy controls [22]. However, reported clinical sEGFR serum concentrations vary greatly depending on detection method and between patients and matched control groups [23]. sEGFR concentrations in cancer patients ranged between 25.5 ± 4.5 (19.4–40.1) in NSCLC vs. 35.9 ± 5.2 (20.6–44.2) ng/mL in controls based upon a commercial ELISA kit (22), to 545 ± 503 (ND-2139) in metastatic breast cancer vs. 546 ± 706 ng/mL (12–3461) in matched controls based upon an acridinium-linked immunosorbent assay (ALISA) assay [24]. In healthy volunteers sEGFR serum concentrations ranged between 0.0125 and 5 μg/mL based upon ALISA [23], corresponding to a 140,000-fold or only 5-fold molar excess of cetuximab at peak and through concentrations compared to sEGFR [30]. In one patient with ovarium cancer up to 9.1 μg/mL sEGFR was measured [20]. It is therefore conceivable that EGFR targeting tracer kinetics could be influenced in a subset of patients with high sEGFR levels. This might be especially true when patient are treatment-naïve, considering many immunoPET agents use microdosing. If patients received multiple doses of EGFR antibody prior to tracer injection, sEGFR could already have been depleted and the sEGFR effect will most likely not be seen.

Strikingly, high liver uptake and rapid blood clearance was shown for ^89^Zr-cetuximab PET in a recent clinical study [17, [37]. ^89^Zr-cetuximab liver radiation burden in patients was up to two-fold higher [34] compared to antibody-based tracers anti-CD20 ^89^Zr-ibritumumab tiuxetan [35] and anti-CD44v6 ^89^Zr-cmAb U36 [36]. Liver uptake of up to 40 mg was also seen for murine cetuximab analog ^111^In-mAb225 in patients, while dose escalation up to 300 mg increased blood availability and tumor uptake of ^111^In-mAb225. [37]. Both mouse and human liver tissues are known to express EGFR [38], although liver uptake in the present study cannot be explained by presence of murine EGFR in liver, as imgatuzumab is not cross-reactive [5]. Our findings suggest that in addition to hepatic EGFR expression, part of the liver accumulation of EGFR antibody tracers in humans might be explained by sEGFR-tracer complexation and subsequent liver sequestration. This would suggest incorporating sEGFR analyses in preclinical and clinical EGFR imaging studies to assess the influence of sEGFR on EGFR tracer kinetics.

Concluding, ^89^Zr-imgatuzumab accumulates in and visualizes EGFR-expressing tumors. High shed sEGFR levels in blood can reduce ^89^Zr-imgatuzumab blood pool availability and lower tumor uptake, due to redirection of tracer-sEGFR complex to the liver. Higher tracer protein dose increased ^89^Zr-imgatuzumab tumor uptake by saturating sEGFR-mediated liver uptake. Combined, these findings could improve interpretation of (pre)clinical EGFR imaging by taking sEGFR levels into account. Furthermore, this phenomenon could possibly also apply to imaging of other antigens with soluble isoforms or which are heavily shed.

## MATERIALS AND METHODS

### Cell Lines

Human cancer cell lines with varying levels of EGFR expression were used. The NSCLC cell lines H441 and A549 and the epidermoid carcinoma cell line A431 were obtained from the American Type Culture Collection (ATCC, Manassas, VA, USA). Cells were quarantined until screening for microbial contamination and mycoplasm was performed and proven to be negative. Cells were tested and authenticated in April 2015 by Baseclear (Leiden, The Netherlands) using STR profiling. Cells were subcultured twice weekly, using Roswell Park Memorial Institute-1640 (RPMI-1640, Gibco, Paisley, UK)/2 mM L-glutamine (Gibco) supplemented with 10% fetal calf serum (FCS, Bodinco, Alkmaar, The Netherlands) for H441, RPMI-1640, with 10% FCS for A549 and high glucose Dulbecco's Modified Eagle's Medium (DMEM, Gibco) with 10% FCS for A431. All cell lines were cultured in a fully humidified atmosphere at 37°C and 5% pCO_2_. Flow cytometric analysis was performed on a BD Accuri™ C6 flow cytometer (BD Biosciences, Breda, The Netherlands) to assess EGFR protein expression status using imgatuzumab (25.3 mg/mL, Roche Glycart AG, Schlieren, Switzerland) as primary antibody and mouse anti-human Fc-specific FITC conjugated secondary antibody (clone HP-6017, Sigma-Aldrich, Zwijndrecht, The Netherlands).

### ^89^Zr-imgatuzumab tracer development and quality control

Imgatuzumab was incubated with a 5-fold molar excess of TFP-N-Suc-desferal-Fe (Df, ABX GmbH, Hamburg, Germany) and subsequent ^89^Zr-labeling was performed as described earlier [8], using clinical grade ^89^Zr (Perkin Elmer, Groningen, The Netherlands). Maximal attainable specific activity was determined using varying amounts of ^89^Zr per mg antibody between 50 and 1000 MBq/mg. Radiochemical purity (RCP) was assessed by trichloroacetic acid (TCA) precipitation test [39]. Df-imgatuzumab conjugates were checked for aggregation and fragmentation by size exclusion high performance liquid chromatography (SE-HPLC). The Waters SE-HPLC system was equipped with a dual wavelength absorbance detector, in-line radioactivity detector and TSK-GEL G3000SWXL column (JSB, Eindhoven, The Netherlands). Phosphate buffered saline (PBS; 140 mmol/l NaCl, 9 mmol/l Na_2_HPO_4_, 1.3 mmol/L NaH_2_PO_4_; pH = 7.4) was used as mobile phase. Stability of ^89^Zr-imgatuzumab was tested for 2 weeks in 0.9% NaCl at 4°C, human serum at 37°C and 0.5 M HEPES pH 7.2 buffer at 37°C.

Immunoreactive fraction of Df-imgatuzumab conjugates was assessed by competition assay. In short, NUNC BreakApart 96-well plates were coated overnight at 4°C with 100 μL 100 ng/mL EGFR ECD (Roche Glycart AG) in 0.1 M Na_2_CO_3_ buffer pH 9.6. ^89^Zr-imgatuzumab was diluted to 4000 ng/mL in assay diluent which consisted out of PBS, 0.5% bovine serum albumin Fraction V and 0.05% Tween 20. Plates were washed with washing buffer (PBS with 0.05% Tween 20) and blocked by 200 μL assay diluent for 1 h at room temperature (RT) and washed again. 0.004 to 156.25-fold molar excess unmodified imgatuzumab was pre-mixed 1:1 with diluted ^89^Zr-imgatuzumab solution and 100 μL of the mixtures were incubated in wells for 1 hour at RT. After washing, wells were broken apart and counted using a calibrated well-type LKB-1282-Compu-gamma system (LKB WALLAC). Counts were plotted against concentration competing unmodified imgatuzumab and the half maximal inhibitory concentration (IC50) was calculated using Graphpad 5.0 (GraphPad Software, Inc., La Jolla, CA, USA). The IC50-value was divided by the final tracer concentration (2000 ng/mL) to yield the immunoreactive fraction.

### Indium-111 labeling IgG control

Human IgG (Nanogam^®^, Sanquin, Amsterdam, The Netherlands) was used as aspecific control. IgG was conjugated with p-SCN-Bn-DTPA (Macrocyclics, Dallas, TX, USA) as described earlier [40]. Radiolabeling was performed using indium-111 (^111^In) chloride (Mallinckrodt, Prague, Czech Republic).

Radiochemical purity of ^111^In-IgG labeling was checked by instant thin layer chromatography (ITLC) using 0.1 M citrate buffer pH 6.0 as eluent.

### Animal studies

Male nude mice (BALB/cOlaHsd-Foxn1^nu^, Harlan, Boxmeer, The Netherlands) were inoculated with A431, H441 (both 5*10^6^ cells in 200 μL PBS) or A549 (3*10^6^ in 300 μL 1:1 PBS and high growth factor Matrigel (BD Biosciences, Breda, The Netherlands) subcutaneously (sc). Xenografts were allowed to grow to at least 100–200 mm^3^. For microPET imaging, A431, A549 and H441 xenograft-bearing mice (*n* = 3–6 per group) were injected intravenously (iv) via the penile vein with ∼10, 25 and 160 μg ^89^Zr-imgatuzumab (effective injected protein doses were 9.6 ± 0.08, 23.8 ± 0.11 and 151.2 ± 1.1 μg, labeled with 4.60 ± 0.06, 3.99 ± 0.13 and 5.64 ± 0.11 MBq ^89^Zr respectively for the 10, 25 and 160 μg dose groups). MicroPET scans were made 1, 3 and 6 days post injection (pi) using a Focus 220 PET scanner (CTI Siemens), followed by *ex vivo* biodistribution analysis after the final scan. Some A431 bearing mice received ∼10, 25 or 100 μg ^89^Zr-imgatuzumab (effective injected protein doses were 9.5 ± 0.05, 23.3 ± 0.26 and 86.0 ± 4.0, labeled with 1.11 ± 0.03, 0.99 ± 0.06 and 0.91 ± 0.09 MBq ^89^Zr respectively for the 10, 25 and 100 μg dose groups) only for *ex vivo* biodistribution analysis 6 days pi. *Ex vivo* results of biodistribution-only A431 tumor bearing mice were pooled with mice used for the microPET imaging study, A431 tumor bearing group size was therefore 6 to 12 animals. All mice received an equal amount of ^111^In-IgG control (1 MBq) as aspecific control. For all tracer injections 10 μg ^89^Zr-imgatuzumab and an equal amount of ^111^In-IgG was used, with cold imgatuzumab and IgG added to reach the total stated protein doses.

Scans were reconstructed and *in vivo* quantification was performed using AMIDE (v1.0.4, Stanford University, Stanford, CA, USA) [41]. MicroPET data are presented as mean standardized uptake value (SUV_mean_). Region of interests (ROI) were drawn for tumor based upon *ex vivo* weight, assuming 1 g/ml tissue density. For blood pool measurements, a fixed-sized sphere was drawn in the center of the heart, for liver and spleen a fixed-sized ellipsoid ROI was drawn in representative parts of the organs. After the final scan, mice were sacrificed and organs of interest collected for biodistribution studies. Organs and standards of the injected tracer were counted in a calibrated well type LKB-1282-Compu-gamma system (LKB WALLAC) and weighed. After decay correction, *ex vivo* tissue activity was expressed as the percentage of injected dose per gram tissue (%ID/g). Xenograft tumors were partly formalin-fixed and paraffin embedded for immunohistochemistry (IHC) and partly frozen for subsequent sEGFR ELISA analysis. Plasma of xenograft and non-xenograft bearing (control) mice was used for sEGFR ELISA analysis, based upon shed c-MET analysis by Jagoda et al. [31]. Plasma was obtained by heart puncture and subsequent centrifugation of blood in lithium heparin blood collection tubes (BD Vacutainer, Plymouth, UK). All animal experiments were approved by the Institutional Animal Care and Use Committee of the University of Groningen.

### sEGFR ELISA analysis

Near-confluent A431, A549 and H441 cell cultures were washed with PBS and incubated for 72 h in fetal calf serum free growth media, as well as serum-containing growth media, as described earlier [26], after which supernates were removed and centrifuged to remove cell debris. Cells of confluent cell cultures were washed with PBS and harvested using trypsin. Lysates of cells, xenograft tumors and liver tissue of A431, A549 and H441 xenograft bearing mice were obtained by mechanical disruption in M-PER Mammalian Protein Extraction Reagent (Thermo Scientific, Bleiswijk, The Netherlands) supplemented with EDTA-free protease/phosphatase inhibitor cocktail (Thermo Scientific). Protein concentrations of cell, tumor and liver lysates were determined by Bradford assay [42]. sEGFR was detected using a human extracellular domain EGFR ELISA (SEK10001, Sino Biological, Beijing, China), according to manufacturer's instructions.

### Immunohistochemistry

Formalin fixed, paraffin-embedded tissue slices were stained for EGFR with a rabbit mAb (clone D38B1 XP^®^, Cell Signaling Technology, Leiden, The Netherlands) and for microvessel density using a rat anti-murine CD31 mAb (clone SZ31, Dianova GmbH, Hamburg, Germany). Hematoxylin & eosin (H&E) staining was performed regularly to assess tissue viability and morphology. Digital scans of slides were acquired by a NanoZoomer 2.0-HT multi slide scanner (Hamamatsu) and analyzed with NanoZoomer Digital Pathology viewer software. A 0–300 EGFR staining histo-score was assessed as follows no staining 0+, weak cytoplasmic staining 1+, strong cytoplasmic staining 2+ and strong membrane and cytoplasmic staining 3+, with H-score calculated using the formula 1 × (% of 1 + cells) + 2 × (% of 2 + cells) + 3 × (% of 3 + cells). Microvessel density was scored in 3 areas, defined as hot spot areas with the maximum number of microvessels as described earlier [39].

### Statistical analysis

Data are presented as mean ± SEM. Statistical analyses were performed using the Mann-Whitney test (Graphpad 5.0) or One-way ANOVA with a Bonferroni post test for multiple comparisons. *P-value*s ≤ 0.05 were considered significant.

## SUPPLEMENTARY MATERIALS FIGURE


