# Supplementary material for: Extracellular domain shedding influences specific tumor uptake and organ distribution of the EGFR PET tracer 89Zr-imgatuzumab
Source: Oncotarget. 2016 Sep 2;7(42):68111–21. doi: 10.18632/oncotarget.11827 (PMC5356542; doi:10.18632/oncotarget.11827)
Supplement: Supplementary file 1 [file oncotarget-07-68111-s001.pdf]

Extracellular domain shedding influences specific tumor uptake and organ distribution of the EGFR PET tracer ^{89}Zr-Imgatuzumab

Supplementary Materials

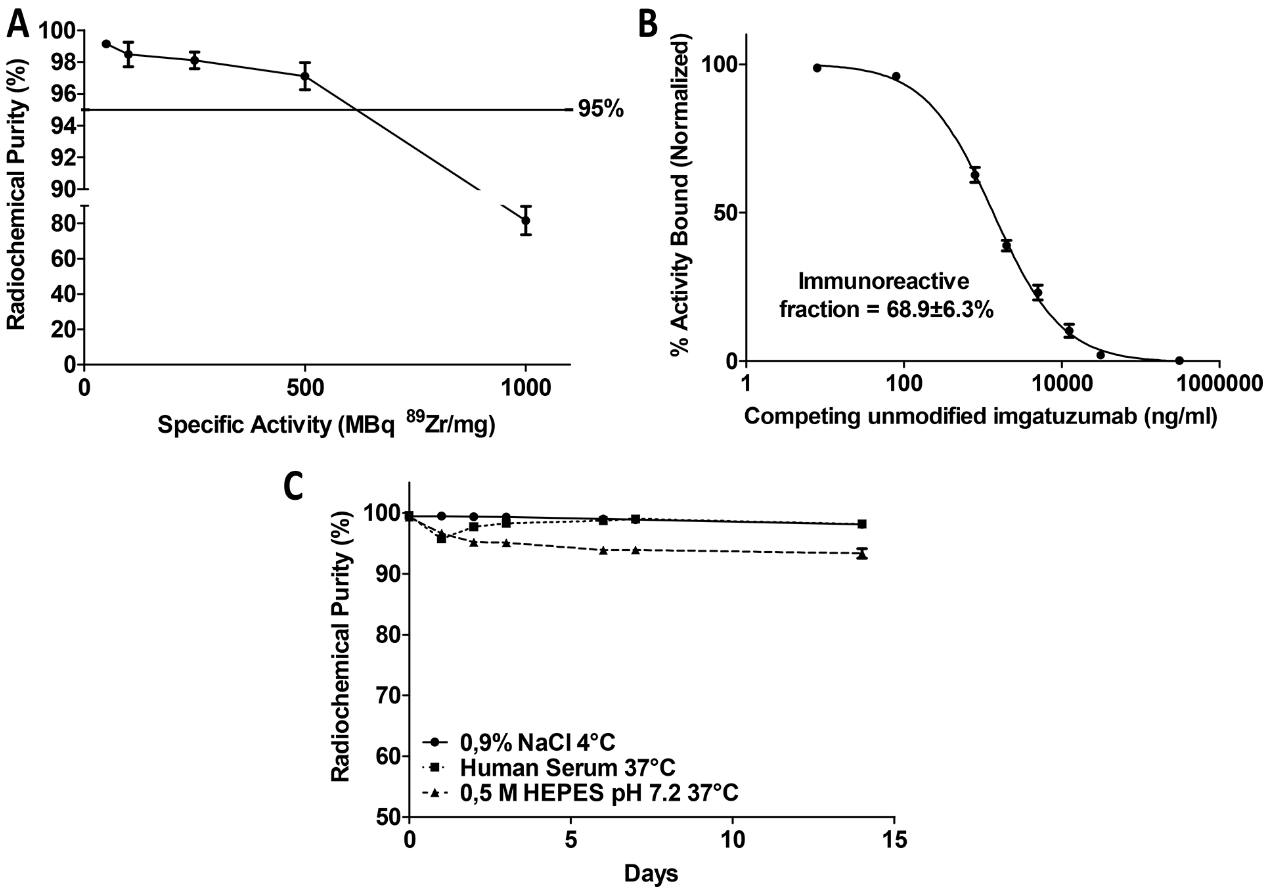

Supplementary Figure S1: (**A**) Maximal attainable specific activity for zirconium-89 labeling of Df-imgatuzumab conjugate. (**B**) Retained affinity of ^{89}Zr-imgatuzumab compared to naked imgatuzumab by competition assay. (**C**) Stability of ^{89}Zr-imgatuzumab in 0.9% NaCl at 4°C, human serum at 37°C and HEPES pH 7.2 at 37° up to 2 weeks.
